# Evaluation of a New CT-Guided Robotic System for Percutaneous Needle Insertion for Thermal Ablation of Liver Tumors: A Prospective Pilot Study

**DOI:** 10.1007/s00270-022-03267-z

**Published:** 2022-09-20

**Authors:** Thierry de Baère, Charles Roux, Frédéric Deschamps, Lambros Tselikas, Boris Guiu

**Affiliations:** 1 BIOTHERIS, Department of Interventional Radiology, Gustave Roussy, Université Paris-Saclay, 114 rue Edouard Vaillant, 94805 Villejuif, France; 2 Department of Radiology, St-Eloi University Hospital, Montpellier, France

**Keywords:** Robotic navigation, Needle navigation, Computed tomography, Liver cancer, Thermal ablation

## Abstract

**Purpose:**

To assess the feasibility and safety of a robotic system for percutaneous needle insertion during thermal ablation of liver tumors.

**Materials and Methods:**

This study analyzed the CT-guided percutaneous needle insertion using the EPIONE robotic device (Quantum Surgical, Montpellier, France) for radiofrequency or microwave liver ablation. The main criteria of the study were feasibility (possibility to perform the thermal ablation after needle insertion), the number of needle adjustments (reiteration of robotically assisted needle insertion when initial needle positioning is considered insufficient to perform ablation), and robotic-guided procedure safety (complications related to the needle insertion). Patients were followed up at 6 months post-intervention to assess local tumor control.

**Results:**

Twenty-one patients with 24 tumors, including 6 HCC and 18 metastases measuring 15.6 ± 7.2 mm, were enrolled. One patient (with one tumor) was excluded for protocol deviation. Robotic assisted thermal ablation was feasible for 22/23 lesions (95.7%) and 19/20 patients (95.0%), as validated by a data safety monitoring Board (95% CI [76.39%; 99.11%]) for the per-protocol population. The mean number of needle adjustments per tumor treated was 0.4 (SD: 0.7), with 70.8% of tumors requiring no adjustment. No adverse events were depicted. Rate of local tumor control was 83.3% for patients and 85.7% for tumors, at 6 months.

**Conclusion:**

This bicentric first-in-human pilot study suggests both feasibility and safety of a stereotactic CT-guided EPIONE device for the percutaneous needle insertion during liver tumor thermal ablation.

## Introduction

Percutaneous thermal ablation is one of the treatments of choice for liver tumors less than 3 cm in size [1, 2]. Radiofrequency ablation (RFA) and microwave ablation (MWA) are the most frequently used modalities and are well documented in the current literature [3, 4]. They have shown favorable outcomes for tumor control, with low associated morbidity, and low local tumor progression (LTP) rate [5]. CT-guided freehand insertion of percutaneous needles sometimes requires numerous CT acquisitions for needle progression control, moreover tumor localization itself and/or poor tumor visibility can lead to a challenging procedure with reduced odds of ablation success.

The development of robotic needle guidance in interventional radiology can help to improve needle insertion, reducing the need for needle adjustments. It also allows the physician to set and visualize the planned ablation margin, to obtain more precise ablation zones with less rates of tumor persistence or recurrence. Lastly, robotic solutions generally offer better positioning results compared with freehand needle insertion [6–10].

We herein report our first in human trial with a CT-guided robotic which is designed to assist interventional radiologists in performing percutaneous procedures in the abdomen. The objective of our work was to assess feasibility and safety for needle insertion.

## Materials & Methods

### Study Design and Patient Selection

This pilot, prospective and bicentric study was approved by the Ethics Committee (Comité de Protection des Personnes, Ile de France V, France) and was registered on ClinicalTrials.gov (NCT04230642).

The inclusion criteria were percutaneous ablation under CT guidance validated by a multidisciplinary tumor board targeting one or two liver tumors in adult patients who have the ability to understand and sign the informed consent.

Exclusion criteria were pregnancy, breast-feeding women, patients unable to undergo general anesthesia, patients unable to tolerate CT contrast agent, or participating to another clinical study.

### Robotically Assisted Percutaneous Thermal Ablation

**Figure 1:**
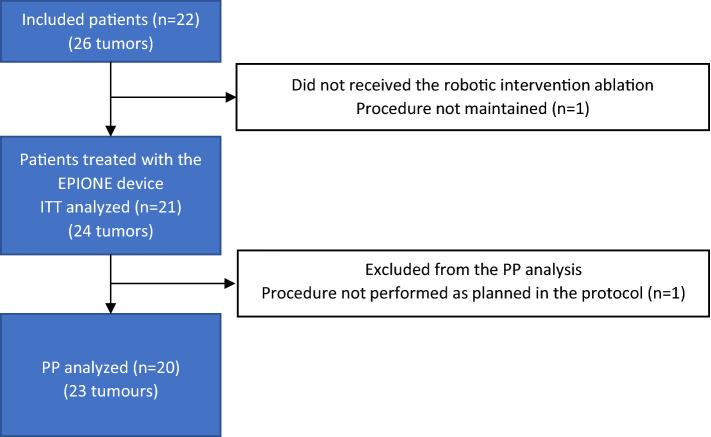
Patient flowchart

Procedures were performed by interventional radiologists with 5 to 25 years of practice in freehand procedures, trained to use the robotic system by carrying out at least one intervention workflow on a phantom model. All interventions were performed under general anesthesia. The choice of the thermal ablation modality was left to the operator’s preference (Fig. 1).

#### Robotic Device

**Figure 2:**
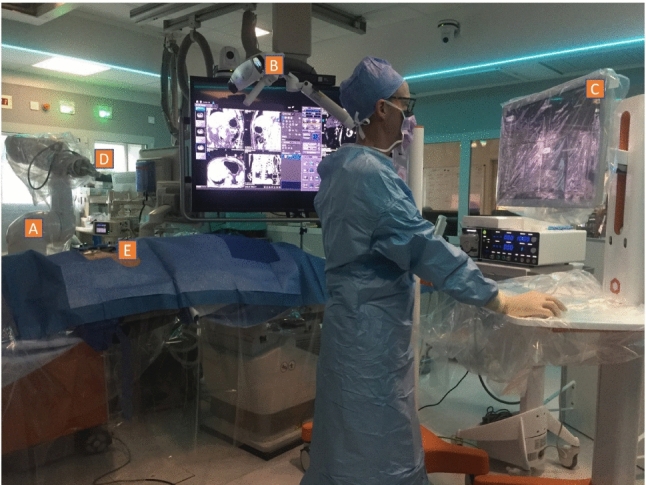
components of the robotic device. The device is composed of a mobile robot (**A**), navigation cart (**B**) and display cart (**C**). Needle guide (**D**) is attached to the robot arm and provides mechanical guidance for rigid straight needles. Patient reference (**E**) is adhesively attached on to the patient’s skin and enables to monitor respiratory motion.

The EPIONE robotic system (Quantum Surgical, Montpellier, France) (Fig. 2) was already detailed in previous animal studies [11, 12]. It is composed of a mobile **display cart** (workstation), a mobile **navigation cart** (infra-red camera), a mobile **robot cart** (robot arm) on which is attached a **needle guide**, and a **patient reference** attached to the patient’s skin.

The planning and navigation software of the device enables to review images, plan a needle trajectory from skin entrance to target, monitor respiratory motion, and automatically position the needle guide according to the trajectory.

**Figure 3:**
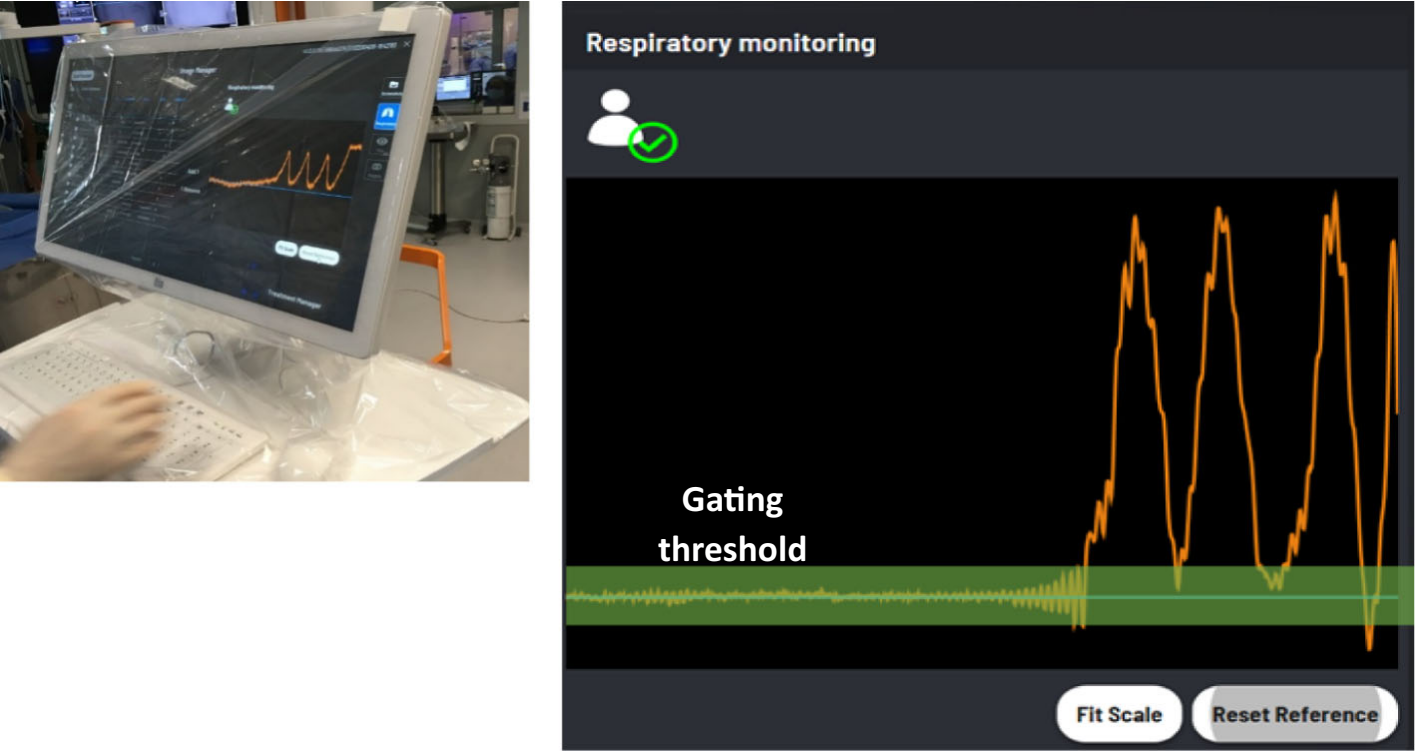
Respiratory monitoring module displayed on the display cart. The orange line is showing the live respiratory movement. When an apnea is performed, the curve stabilizes, and a reference level can be defined (blue line). A gating threshold is also displayed as a green gating band to help verifying apnea or breath-hold repeatability.

Using a 3D motion analysis of the patient reference, the respiratory monitoring module enables real-time tracking and display of the patient’s respiratory cycle (Fig. 3), allowing the operator to visually check the breathing phase and repeatability of apnea to record a respiratory reference level.

The registration between the patient position on the table, the CT images and the robotic device are automatically performed by the system using the patient reference.

#### Robotic Needle Insertion Planning

**Figure 4:**
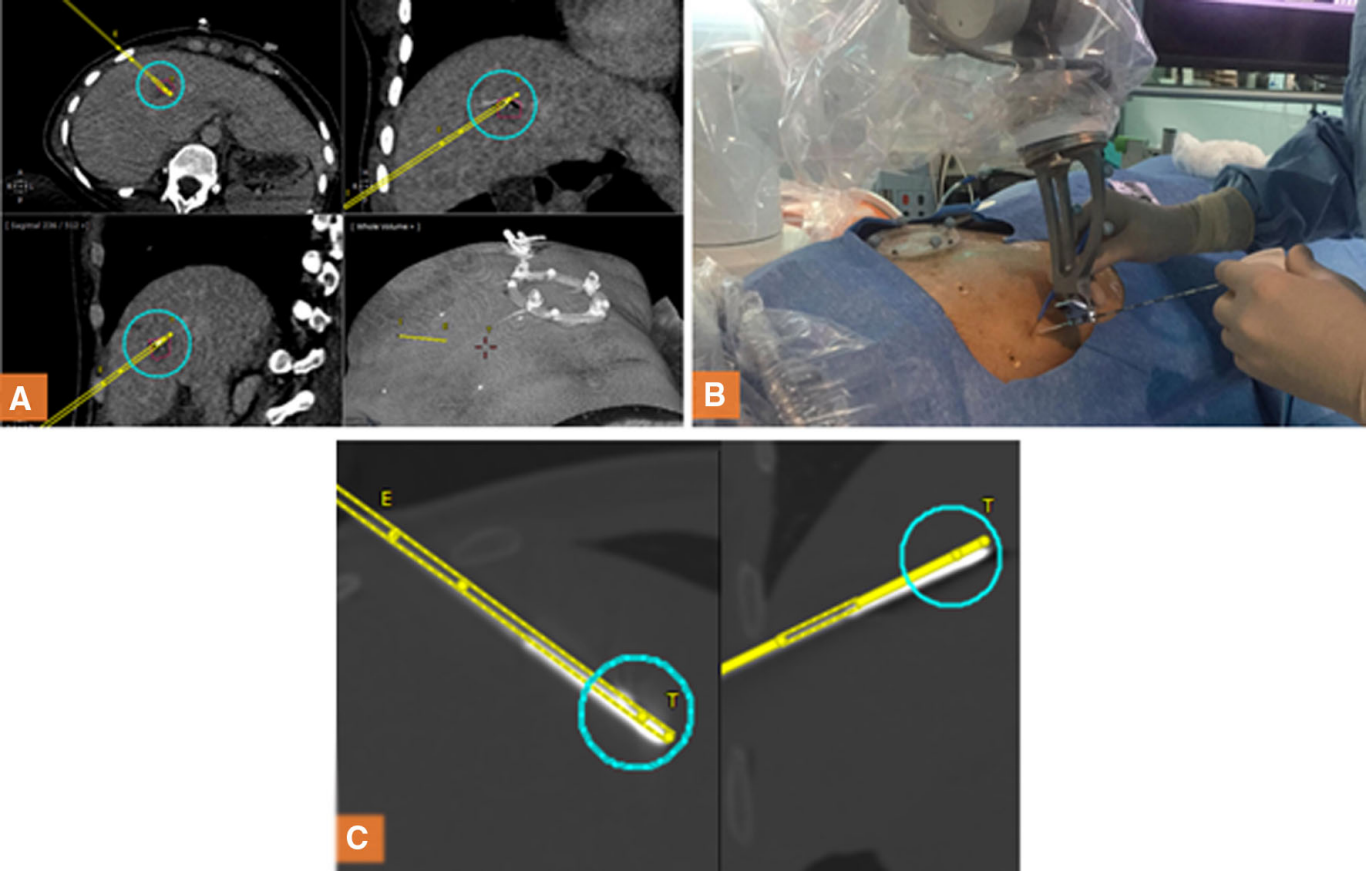
Example of robot-guided needle placement for thermal ablation (**A**). Planning definition on CT-scan image of needle trajectory (in yellow) to treat a lesion of segment IVa (segmented in red). The simulation of the ablation zone (in blue) is positioned to entirely cover the lesion and safety margins. (**B**) Robotic guidance: robot automatic positioning to align needle guide on planned trajectory and needle insertion in single-pass through needle guide until the mechanical stop. (**C**) Needle placement control: after insertion, the needle placement is compared with the planned trajectory and the ablation is performed if the needle placement is satisfying.

A baseline CT was acquired under apnea using an Alphenix or Infinix-i CT scanner (Canon Medical Systems, Otawara, Japan) and then loaded in the software of the robotic system. The type and length of the ablation needle were entered into the planning software. The interventional radiologist defined the target point so that the expected ablation zone covers the tumor and the entry point at the needle puncture site on skin surface. The needle trajectory path was then calculated by the device software and shown on the display screen (Fig. 4, image A). The needle trajectory was carefully confirmed by the operator being sure to avoid critical or bone structures and sent to the robotic arm for execution.

#### Robotic-Guided Needle Insertion

The robotic arm was manually pre-positioned close to the patient’s abdomen with a footswitch command control. Under apnea, the operator inserted the needle through the needle guide until the end stop was reached (Fig. 4, image B). The needle was then released from the needle guide and the robot arm was manually withdrawn. A post-insertion CT-scan image was performed to assess the feasibility (possibility to perform the procedure related to the needle positioned by the robot) (Fig. 4, image C).

#### Post-operative Assessment and Ablation Success

A CT-scan acquisition was performed at the end of the intervention to ensure the absence of complication, and to assess whether the tumor was completely ablated or not. The patients were then reviewed periodically every 3 months using either CT-scan or MRI to assess LTP, as per standard practice guidelines [13]. The follow-up performed after a minimal 6-month period was to collect data to assess the local tumor control rate, as routine follow-up.

### Outcome Measures

#### Ablation Feasibility

Feasibility was defined as a needle placement judged by the operator as adequate to perform ablation. The operator visually estimated on the post-insertion CT-scan if the needle was positioned accurately enough compared to the planned trajectory. To confirm feasibility assessment, the post-insertion CT-scan was ultimately reviewed by the Data and Safety Monitoring Board (DSMB), constituted of 2 experts in interventional radiology and one expert in digestive surgery who worked in different institutions.

#### Needle Placement Adjustment

If the ablation was not considered feasible following the first needle insertion, the operator may choose to adjust the position by planning a new trajectory. The number of needle placement adjustments (attempts required to reach a position that met the feasibility criteria) was recorded. After a second failed adjustment attempt, the procedure was considered unsuccessful and converted into a freehand insertion.

#### Safety

Safety was evaluated as the number of major adverse events attributable to the needle insertion are reported using the Society of Interventional Radiology (SIR) scale [14, 15] and the Cardiovascular and Interventional Radiological Society of Europe (CIRSE) classification [16]. The adverse events were monitored and reviewed by the DSMB to confirm the safety evaluation by the operator.

### Statistical Analysis

All statistical analyses were performed using SAS® 9.4 software (Cary, NC, USA). Description of relevant study variables was: *n* (total number of data), number of missing data, mean, standard deviation (SD), median, min and max for quantitative variables and using number of missing data, frequency and percentage of non-missing for qualitative variables. The statistical analysis took into account data clustering (several ablations for a given patient) in case of inferential statistics. Data were analyzed according to both the intention-to-treat (ITT) and per protocol (PP) principles.

## Results

### Patient Flow and Baseline Data

From May 2020 to January 2021, 22 patients were enrolled with 26 liver tumors. Patients were either treated at the institution A (*n* = 16) or B (*n* = 6) (Fig. 1). During the study period, one patient (with two tumors) for which the ablation procedure was not maintained was considered as a screening failure and was therefore excluded from the study. Of the 21 patients with 24 tumors remaining in the ITT population, one patient (with one tumor) was excluded from the PP analysis due to protocol deviation (the CT-scan was performed later than planned in the protocol).

Baseline characteristics of the 21 patients (9 women, 12 men) of ITT analysis are given in Table 1. Patients had a mean body mass index of 23.8 ± 5. kg/m^2^ and a mean age of 64.8 ± 11.9 years. The liver targeted tumors were BCLC stages A hepatocellular carcinomas in 5 (23.8%) patients and metastases in 16 (76.2%) patients. The mean largest diameter of the tumors was 15.6 ± 7.2 mm (range 5–32 mm). Eleven (45.8%) targeted tumors were judged as challenging by the operators in regard with their location including 9 (81.8%) lesions located in the hepatic dome and two (18.2%) subcapsular lesions. Eighteen (75%) trajectories were not in the axial plane and fifteen (62.5%) trajectories had a double angulation (i.e., craniocaudal and lateral).

**Table 1:** Baseline characteristics

Parameter	*N* (%)	Mean (SD)	Min; Max
Demographics			
Age of consent (years old)		64.8 (11.9)	35.0; 79.5
Gender			
Male	12 (57.1)		
Female	9 (42.9)	23.8 (5.8)	15.6; 40.5
BMI (kg/m^2^)			
Targeted lesion			
Lesion entity			
Hepatocellular carcinoma	5 (23.8)		
Liver metastases	16 (76.2)		
Number of liver lesion(s) per patient			
1	18 (85.7)		
2	3 (14.3)		
Total number of lesions	24		
Lesion Size (diameter in mm)	–	15.6 (7.2)	5.0; 32.0
Lesion location	2 (8.3)		
Segment II	1 (4.2)		
Segments II VIII	2 (8.3)		
Segment III	3 (12.5)		
Segments IV V	5 (20.8)		
Segments V VI	1 (4.2)		
Segment VI	2 (8.3)		
Segment VII	4 (16.7)		
Segment VIII	4 (16.7)		
Challenging lesions	_		
No	13 (54.2)		
Yes	11 (45.8)		
Lesion located in hepatic dome	9 (85.9)		
Subcapsular lesion	2 (18.2)		
Needle placement			
Trajectory			
Axial (in plane)	6 (25.0)		
Oblique (out of plane)	18 (75.0)		
Angulations			
Lateral (°)	_		– 33.5; 78.8
Cranio-caudal (°)	_		– 16.9; 61.4
Needle type			
Dophi (Surgnova)	18 (75.0)		
Octopus (Starmed)	1 (4.2)		
Solero (Angiodynamics)	5 (20.8)		
Needle length			
* 150mm*	9 (37.5)		
* 190mm*	5 (20.8)		
* 200mm*	10 (41.7)		
Needle diameter			
15G	23 (95.8)		
17G	1 (4.2)		
Ablation parameters per lesion			
Type of thermal ablation			
Radiofrequency	1 (4.2)		
Microwave	23 (95.8)		
Cumulative ablation time (min)	_	7.5 (5.7)	3.0; 31.0
Cumulative power for microwave ablation (watts)		81.3 (32.2)	50.0; 160.0
Operator			
≤10 years of experience			
Patients treated	6 (28.6)		
Lesions treated	7 (29.2)		
>10 years of experience			
Patients treated	15 (71.4)		
Lesions treated	17 (70.8)		

Min; Max, Minimum; Maximum

SD, Standard Deviation

MWA was used in 23 (95.8%) tumors, using a 15G Dophi (Surgnova, HDTech, Lorient, France) needle in 18 (75%) tumors and a 15G Solero (Angiodynamics, New York, USA) needle in 5 (20.8%) tumors. RFA was used in one (4.2%) tumor with a 17G Octopus (Starmed, Gyeonggi-do, South Korea) needle. The needle length was 150mm in 9 (37.5%) lesions, 190mm in 5 (20.8%) and 200mm in 10 (41.7%).

All 24 tumors were treated by 4 operators. Seventeen (70.8%) lesions in 15 (71.4%) patients were treated by 2 senior operators having more than 10 years of experience, the remaining 7 (29.2%) lesions in 6 (28.6%) patients were treated by 2 less experienced operators.

The mean (SD) overall procedure duration from first pre-interventional CT-scan to last post-ablation CT-scan was 73.8 (29.2) min.

### Feasibility

Among the total number of 23 ablations included in the PP analysis, the robotic needle placement was judged adequate to perform ablation for 22 lesions, giving a 95.7% feasibility rate (Table 2), confirmed by the DSMB blind review. The unique failure was recorded when the initially planned needle trajectory was not accessible with the robot arm. After planning a new trajectory, insertion of the ablation needle resulted in a sliding of the needle on the liver’s capsule surface, and the procedure was ultimately resumed freehand under CT guidance.

**Table 2:** Number of patients with no major AE related to the CT-guided ablation procedure AND with the needle positioned accurately enough to allow the next step of the procedure to be performed by patient and by lesion (PP population)

Parameter	*N* (%)	95 CI
Combined “feasibility” and “no major Adverse Event”		
By lesion	23 (100)	65.9%-99.6%
Yes	22 (95.7)	
No	1 (4.3)	
Concordance of response between operator and DSMB related to feasibility		
Both “yes”	22 (100)	
Both “No”	1 (100)	
Investigator “No” and DSMB “Yes”	0	
Investigator “Yes” and DSMB “No”	0	

DSMB, Data Safety Monitoring Board

95 CI, Confidence Interval at 95%

### Needle Placement Adjustment

No needle placement adjustment was needed in 17 (70.8%) tumors, while 6 (25%) lesions required 1 adjustment and 1 (4.2%) lesion required 3 adjustments. The mean number of adjustments per lesion was 0.4 ± 0.7 (Table 3).

**Table 3:** Number of needle placement re-adjustments

Parameter	*N* (%)	Mean (SD)	Median (Q1; Q3)
Number of needle adjustments			
By lesion	24 (100)	0.4 (0.7)	0.0 (0.0; 1.0)
0	17 (70.8)		
1	6 (25.0)		
2	–		
3	1 (4.2)		

Q1; Q3: Inferior Quartile; Superior Quartile

SD, Standard Deviation

### Safety

No procedure-related complications were observed on post-procedural CT-scan, and no adverse events were detected. Twenty patients were discharged from hospital the day after the procedure, one patient after 2 days.

At 6 months of follow-up, two patients died before review, determining an overall survival of 90.0% in the PP population, results are reported in Table 4. The causes of death were not related to the ablation procedure or the robot use, but to cancer progression.

**Table 4:** Survival rates at 6 months follow-up in the PP population

Parameters at 6 months	Patients	Lesions
Overall survival	18/20 (90.0%)	21/23 (91.3%)
Disease free survival	15/18 (83.3%)	18/21 (85.7%)

### Ablation Success

Among the 18 patients with 21 lesions that were followed up to 6 months, a local tumor control was obtained in 15 (83.3%) patients and 21 (85.7%) tumors, three patients have developed LTP and were retreated with MWA.

## Discussion

No adverse events were reported in this study in relation with the use of the device, confirming the safety data collected in animal pre-clinical studies [11, 12]. Only few other devices are available for robotic needle placements, and no specific complications were reported in prospective clinical investigations involving these devices [3, 7, 17–19]. The short hospital stay with quick recovery of our patients is similar to results reported in the literature on navigational devices for liver ablations [10, 20–23].

In our cohort, feasibility was achieved for 95.7% of the lesions. This result is consistent with the rate of 89% in 28 lesions reported by Engstrand et al. with another navigation device [20]. In our PP population, we observed one case of non-feasibility, whereas the first trajectory could not be reached by the robot arm which was stopped before a possible contact with the patient, the second attempt resulted in the sliding of the needle along the liver capsule due to a steep approach angle. Therefore, in future it would be relevant to plan trajectories as perpendicular as possible to the liver capsule. In our study, the mean number of needle adjustment per lesion was 0.4 (± 0.7) and 29.2% of lesions requiring needle adjustment. It is lower than rates from other reports of robotic needle placement with manual repositioning required in 48.7% to 60% of the interventions, and a mean number of adjustment from 0.8 (± 0.8) to 1.1 (± 0.7) per lesion [7, 17]. Needle placement has a major impact on the treatment outcomes since any adjustment may increase radiation dose, procedure time, tissue trauma, and possibly the risk of bleeding and tumor seeding; but moreover approximative needle placement will decrease the probability of local tumor control [20, 22, 24, 25].

**Figure 5:**
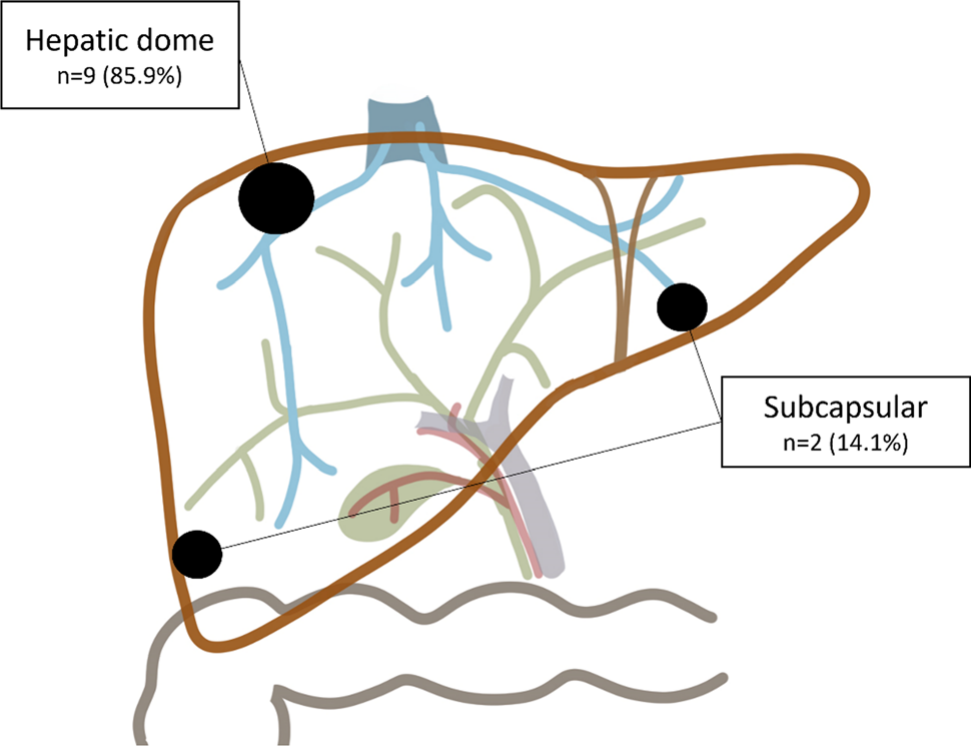
Challenging liver tumors localization.

The targeting potentialities are highly dependent on tumor visibility and location of the lesion within the liver, with a third of lesions judged “unablatable” in the clinical setting [21, 26]. We reported that 46% of our patients had tumors judged as challenging to target (Fig. 5), but tumor location did not influence feasibility. Lachenmayer et al. [21] and Tinguely et al. [22] using a CT-guided percutaneous system, and Odisio et al. [27] using freehand insertion under CT guidance for liver ablations recently reported similar results. Tinguely et al. showed complex targeting trajectories such as intercostal trajectories and steep trajectory angles have no impact on targeting accuracy [22]. In our study, eighteen (75%) trajectories were out of plane with a minimum and maximum craniocaudal angulations from − 16.9 to 61.4°, demonstrating the ability of the device to target lesions requiring significant angulation without impacting feasibility.

There are several limitations and potential shortcomings to the present study. First and foremost, this is not a comparative and randomized trial. We chose to design a single-arm study to collect the first data in Human with a new robotic device. A simple design was therefore preferred to demonstrate the main outcomes which were feasibility and safety. Additionally, the number of patients and lesions may be too small to allow robust statistical analyses and identify differences in our results compared to other data from the literature. Furthermore, the radiation dose received by the patient was not analyzed in the present study although the low rate of needle adjustment demonstrated in this study highlights the fact that the majority of patients had only 3 CT-scan acquisitions (treatment planning CT, needle placement assessment CT, and post-ablation CT), and no radiation was delivered to the operator. These could be interesting parameter to evaluate in the future.

## Conclusion

Robotic arm assistance for needle positioning during thermal ablation was safe and allowed a single insertion in 70.8% of targeted tumors, with an overall mean number of adjustments of 0.4 ± 0.7 per lesion.

Our present first-in-human limited series suggests both feasibility and safety of a stereotactic CT-guided device for the percutaneous needle insertion for liver tumors treatment. Other CT-guided interventions such as biopsies and other organs in abdomen might benefit from such needle guidance.
